# Assessment of Morphological Variations of the Coronoid Process, Condyle, and Sigmoid Notch as an Adjunct in Personal Identification Using Orthopantomograms Among the North Indian Population

**DOI:** 10.7759/cureus.40275

**Published:** 2023-06-11

**Authors:** Sandeep K Bains, Archana Bhatia, Nishant Kumar, Aman Kataria, Isha Balmuchu, Sanjeeta Srivastava

**Affiliations:** 1 Department of Oral Medicine and Radiology, Surendera Dental College and Research Institute, Sri Ganganagar, IND; 2 Department of Periodontology and Implantology, Surendera Dental College and Research Institute, Sri Ganganagar, IND

**Keywords:** orthopantomograms, panoramic radiograph, sigmoid notch, condyle, coronoid process

## Abstract

Aim: The aim of this study is to assess morphological variations of the coronoid process, condyle, and sigmoid notch as an adjunct in personal identification using orthopantomograms among the North Indian population.

Methodology: The study sample (n=240) was distributed into four age groups: Group I: 30 males and 30 females aged 10-19 years, Group II: 30 males and 30 females aged 20-29 years, Group III: 30 males and 30 females aged 30-39 years, and Group IV: 30 males and 30 females aged 40-59 years. All were subjected to panoramic radiographs. The different morphological forms of the coronoid process, condyle, and sigmoid notch were evaluated.

Results: The results showed that across all age groups, angular condyles were the most common kind of condyle in males, followed by round and convex types. The present study found that the coronoid process typically takes on a triangle shape across all ages and sexes. Additionally, the vast majority of cases were triangular on both sides, and this was true across both sexes. It was found in this study that the sigmoid notch most commonly took the form of a larger notch, followed by a rounder notch.

Conclusion: Using panoramic photos to portray the different morphologies of the coronoid process, condyle, and sigmoid notch can be a much simpler and faster method of identifying an individual, especially in the event of a mass disaster, so long as antemortem data are kept. The method of radiographic identification of individuals has recently gained prominence due to its efficacy. Radiographs like these can be invaluable in forensic dentistry, where they can help unearth previously hidden evidence if premortem records are retained. As a potential approach for individual identification among our population, panoramic radiographs were used to investigate the varying morphological forms of the coronoid process, condyle, and sigmoid notch.

## Introduction

Numerous anthropological research studies using dry skulls have been carried out for decades in an effort to identify the deceased [1]. One of the most reliable and quick methods used for human identification in forensics is the comparison of antemortem and postmortem radiographs. Identification of a person has always been necessary for a number of personal, civil, and legal issues related to a person's identification, as well as for the certification of death. For many years, fingerprint analysis and DNA matching have made it easier to identify a person. However, when the carcass is completely charred and reduced to skeletal remains, human identification actually becomes challenging and difficult. In these situations, anthropological and dental analyses are crucial for establishing the person's identity [1].

The mandible is the strongest and most substantial of the human facial bones. As essential tools in anthropological and forensic investigations, several morphological variants of the coronoid process, condyle, and sigmoid notch have already been described in the literature. These variances might be hereditary in nature or result from functional adjustments brought on by the growing process [2].

One of the most reliable and quick methods used for human identification in forensics is the comparison of antemortem and postmortem radiographs. In forensic dentistry, radiographs are crucial for revealing concealed information that is difficult to see during a routine physical examination [3]. The most commonly recommended radiographs in dentistry for diagnosis and tracking the prognosis of various treatment treatments are panoramic images. Additionally, it has been said that panoramic radiography is trustworthy for portraying the condyle and assessing the abnormalities of the temporomandibular joint. Moreover, when associated with more modern imaging modalities like computed tomography (CT), magnetic resonance imaging (MRI), and cone beam computed tomography (CBCT), maxillofacial radiography using an orthopantomogram (OPG) is reported to be less costly and is utilized as a standard screening tool in numerous sectors of dentistry [4]. We performed this study to assess the morphological variations of the coronoid process, condyle, and sigmoid notch as an adjunct in personal identification among the population of Sri Ganganagar using OPGs.

## Materials and methods

The prospective study was conducted on randomly selected OPGs of the patients (n=240) reporting to daily OPD, Department of Oral Medicine and Radiology, Surendera Dental College and Research Institute, Sri Ganganagar, Rajasthan aged between 10 and 60 years. The patients requiring digital OPGs for diagnostic, periodontal, surgical (for impacted teeth), mixed dentition status or orthodontic purposes were recruited for the study. All the patients were briefed about the study and written informed consent in both English and local language was obtained from everyone. Institutional ethical approval was obtained to carry out the study with letter number "SDCRI/IEC/2020/006". The subjects were selected based on the following criteria. Inclusion criteria were OPGs of patients with an age range of 10-60 years (including both genders) and OPGs with clearly visible condyle, coronoid, and sigmoidal notch of mandible bilaterally. Exclusion criteria were completely edentulous patients, a history of trauma, orthognathic surgeries, patients with pain, swelling, trismus, or clicking sounds over TMJs, patients with any developmental or acquired pathology affecting mandibular condylar, coronoid, or sigmoid notch morphology, patients with systemic, inflammatory, or endocrinal disorders affecting the development of mandibular condyle, coronoid or sigmoid notch morphology, and uncooperative patients.

**Figure 1:**
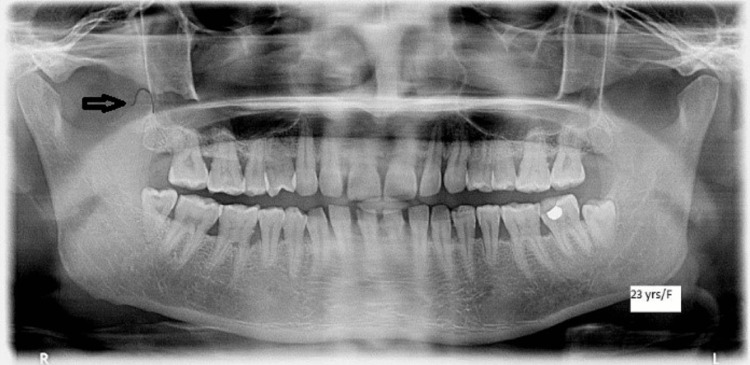
Orthopantomagram showing the coronoid process The black arrow shows the coronoid process

**Figure 2:**
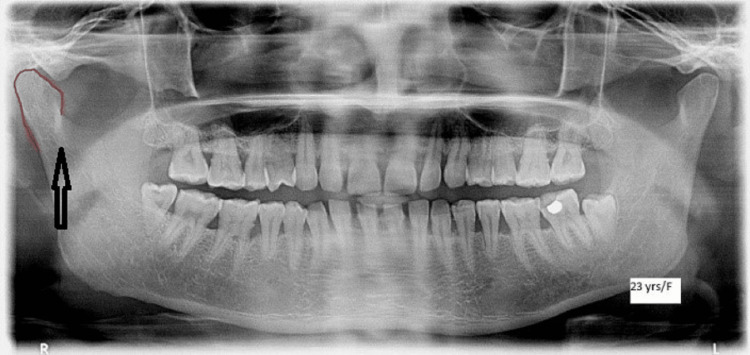
Orthopantomagram showing the condyle The black arrow shows the condyle

**Figure 3:**
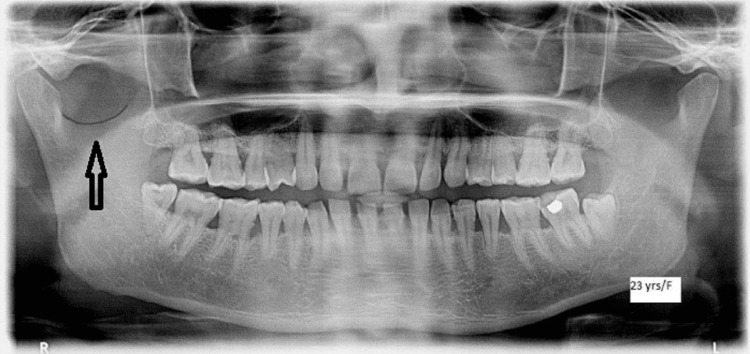
Orthopantomagram showing the sigmoid notch The black arrow shows the sigmoid notch

The study sample was distributed into four age groups. A convenience sampling method was used. The sample size was calculated using n = Z^2^pq/d^2^, where Z = 1.96 at 95% confidence interval and p = 55.4%, and d = 6% margin of error [5]. The total sample size was calculated to be 210.96. However, we included 240 samples in our study: Group I: 30 males and 30 females aged 10-19 years, Group II: 30 males and 30 females aged 20-29 years, Group III: 30 males and 30 females aged 30-39 years, and Group IV: 30 males and 30 females aged 40-59 years. All patients were assessed clinically in a dental chair and were subjected to rinse with 0.2% chlorhexidine-gluconate mouthwash. Demographic details (age, gender, diet) and medical/dental history were recorded. Patients who met the selection criteria were examined clinically before undergoing digital panoramic radiography using a Kodak Carestream CS8000C OPG/Cephalometric system (Carestream Health, Inc., Rochester, New York, USA) with the manufacturer-recommended exposure parameters. The different morphological forms of the coronoid process, condyle, and sigmoid notch were evaluated (Figures 1-3). Right and left coronoid process and sigmoid notch shapes and condyle shapes were recorded and were interpreted according to Shakya et al. and Hegde et al., respectively [5,6]. Shapes of the coronoid process were recorded as triangular, round, beak, and flat. Shapes of the sigmoidal notch of the mandible were recorded as round, sloping, and wide. Shapes of the mandibular condyle from all the 240 OPGs were assessed by two oral and maxillofacial radiologists independently for preventing inter-observer bias. The data thus collected will be tabulated and subjected to further statistical analysis using IBM SPSS Statistics for Windows, Version 18 (Released 2009; IBM Corp., Armonk, New York, United States). The p value less than 0.05 was set as significant.

## Results

In the age group of 10-19 years, the most common condylar type was angled seen in 33 males and 16 females, followed by a round in 17 males and 15 females and convex in 10 males and nine females. There was no statistically significant difference in the left and right sides of both genders (P>0.05) (Table 1).

**Table 1:** Gender and side-wise distribution of condylar types in the age group of 10-19 years

Gender	Side	Angled		Convex		Round		Chi square value	p value
		Frequency	Percentage	Frequency	Percentage	Frequency	Percentage		
Male	Left	14	46.7	6	20	10	33.3	1.68	0.43
	Right	19	63.3	4	13.3	7	23.3		
Female	Left	7	23.3	2	6.7	1	3.3	5.72	0.06
	Right	9	30	7	23.3	14	46.7		

In the age group of 10-19 years, the most common coronoid type identified was triangular seen in 36 males and 37 females, followed by round in 11 males and 13 females, flat in 10 males and four females, and break in three males and six females. The difference was non-significant in both sides of both genders (P> 0.05) (Table 2). In the age group of 10-19 years, the most common sigmoid notch type was widely seen in 27 males and 14 females. The difference was non-significant in both sides of both genders (P>0.05) (Table 3).

**Table 2:** Gender and side-wise distribution of coronoid types in the age group of 10-19 years

Gender	Side	Beak		Flat		Round		Triangular		Chi square value	p value
		Frequency	Percentage	Frequency	Percentage	Frequency	Percentage	Frequency	Percentage		
Male	Left	1	3.3	4	13.3	7	23.3	18	60	1.55	0.67
	Right	2	6.7	6	20	4	13.3	18	60		
Female	Left	4	13.3	3	10	5	16.6	18	60	2.38	0.49
	Right	2	6.7	1	3.3	8	26.7	19	63.3		

**Table 3:** Gender and side-wise distribution of sigmoid notch types in the age group of 10-19 years

Gender	Side	Round		Sloping		Wide		Chi square value	p value
		Frequency	Percentage	Frequency	Percentage	Frequency	Percentage		
Male	Left	9	30	9	30	12	40	0.642	0.72
	Right	7	23.3	8	26.7	15	50		
Female	Left	16	53.3	8	26.7	6	20	1.73	0.42
	Right	18	60	4	13.3	8	26.7		

In the age group of 20-29 years, the most common condylar type was angles seen in 37 males and 29 females followed by a round in 17 males and 23 females, convex in three males and seven females, and flat in three males and one female. The difference was non-significant in both sides of both genders (P>0.05) (Table 4). The most common coronoid type was triangular seen in 40 males and 37 females followed by flat type in nine males and 10 females, break in eight males and nine females, and round in three males and nine females. The difference was non-significant in both sides of both genders (P> 0.05) (Table 5). In the age group of 20-29 years, the most common sigmoid notch was widely seen in 31 males and 34 females followed by a round in 24 males and 13 females and sloping in five males and 13 females. The difference was non-significant in both sides of both genders (P> 0.05) (Table 6).

**Table 4:** Gender and side-wise distribution of condylar types in the age group of 20-29 years

Gender	Side	Angled		Convex		Flat		Round		Chi square value	p value
		Frequency	Percentage	Frequency	Percentage	Frequency	Percentage	Frequency	Percentage		
Male	Left	19	63.3	2	6.7	1	3.3	8	26.7	0.75	0.86
	Right	18	60	1	3.3	2	6.7	9	30		
Female	Left	15	50	3	10	1	3.3	11	36.7	1.22	0.74
	Right	14	46.7	4	13.3	0	0.0	12	40		

**Table 5:** Gender and side-wise distribution of coronoid types in the age group of 20-29 years

Gender	Side	Beak		Flat		Round		Triangular		Chi square value	p value
		Frequency	Percentage	Frequency	Percentage	Frequency	Percentage	Frequency	Percentage		
Male	Left	2	6.7	3	10	3	10	22	73.3	6.4	0.09
	Right	6	20	6	20	0	0.0	18	60		
Female	Left	3	10	4	13.3	3	10	20	66.7	1.24	0.74
	Right	3	10	4	13.3	6	20	17	56.7		

**Table 6:** Gender and side-wise distribution of sigmoid notch types in the age group of 20-29 years

Gender	Side	Round		Sloping		Wide		Chi square value	p value
		Frequency	Percentage	Frequency	Percentage	Frequency	Percentage		
Male	Left	14	46.7	2	6.7	14	46.7	1.15	0.56
	Right	10	33.3	3	10	17	56.7		
Female	Left	4	13.3	6	20	20	66.7	3.05	0.21
	Right	9	30	7	23.3	14	46.7		

**Figure 4:**
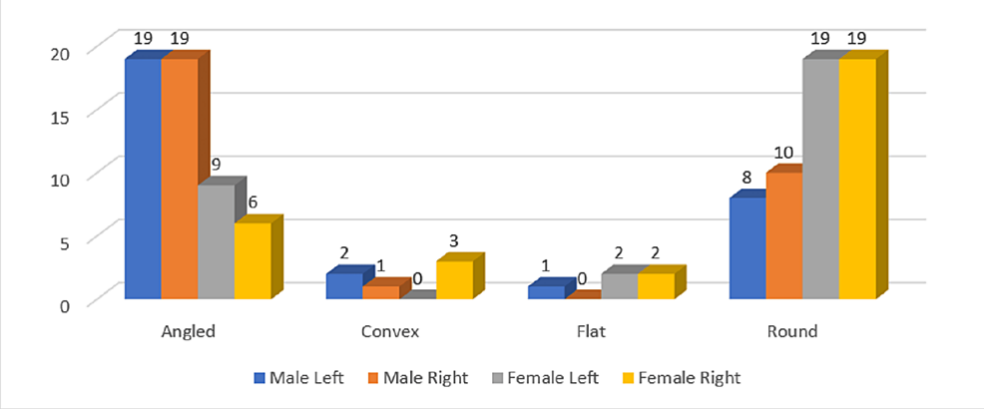
Gender and side-wise distribution of condylar types in the age group of 30-39 years

**Figure 5:**
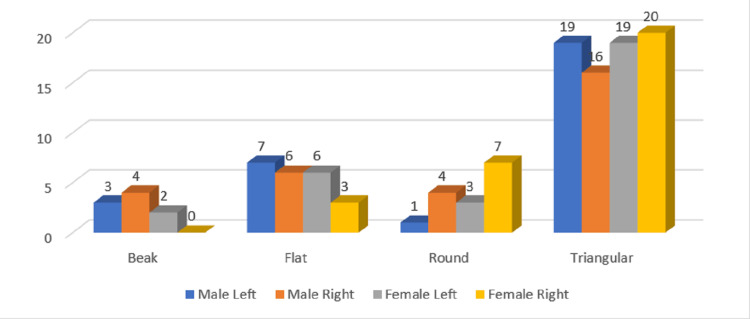
Gender and side-wise distribution of coronoid types in the age group of 30-39 years

**Figure 6:**
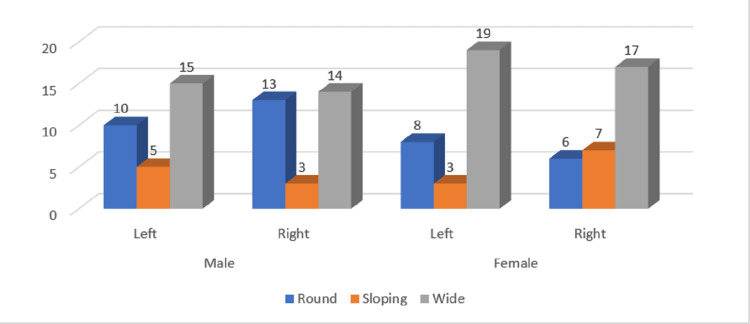
Gender and side-wise distribution of sigmoid notch types in the age group of 30-39 years

In the age group of 30-39 years, the most common condylar type was "angled" angle seen in 38 males and 15 females followed by round in 18 males and 38 females. The difference was non-significant in both sides of both genders (P> 0.05) (Figure 4). The most common coronoid type in the age group of 30-39 years was the triangular type seen in 365 males and 39 females. The difference was non-significant in both sides of both genders (P> 0.05) (Figure 5). The most common sigmoid type among the 30-39 years age group was wide type seen in 29 males and 36 females. The difference was non-significant in both sides of both genders (P> 0.05) (Figure 6).

**Figure 7:**
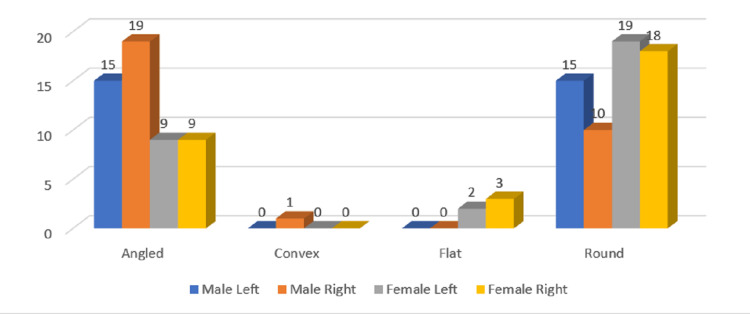
Gender and side-wise distribution of condylar types in the age group of 40-59 years

**Figure 8:**
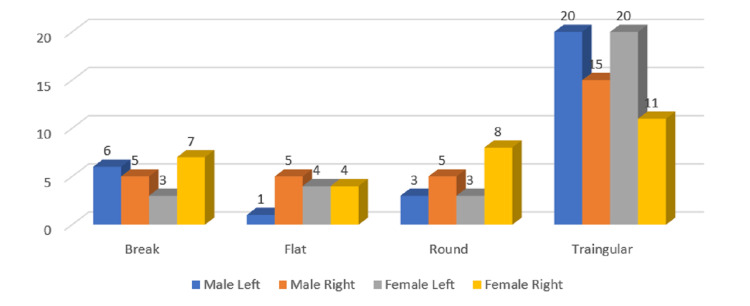
Gender and side-wise distribution of coronoid types in the age group of 40-59 years

**Figure 9:**
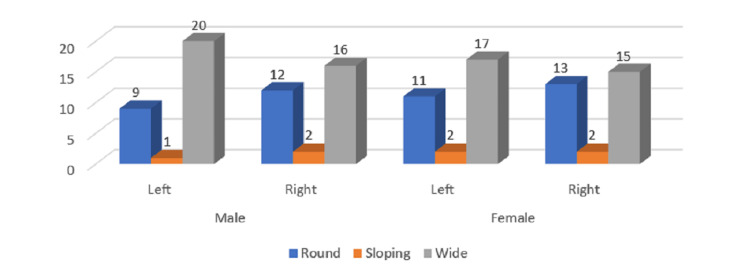
Gender and side-wise distribution of sigmoid notch types in the age group of 40-59 years

In the age group of 40-59 years, the most common condylar type was the round type seen in 25 males and 37 females. The difference was non-significant in both sides of both genders (P>0.05) (Figure 7). The most common coronoid type in the age group of 40-59 years was the triangular type seen in 35 males and 31 females. The difference was non-significant in both sides of both genders (P>0.05) (Figure 8). The most common sigmoid notch types in the age group of 40-59 years were the wide type seen in 36 males and 32 females. The difference was non-significant in both sides of both genders (P> 0.05) (Figure 9).

## Discussion

Anthropologists have studied dry skulls extensively for decades in an effort to identify the deceased. Comparing antemortem and postmortem radiographs is one of the quickest and most accurate human identification methods used in forensics [7]. Personal, civil, and legal matters, as well as the certification of a person's death, have always required the identification of the individual in question. Fingerprinting and DNA matching have been used to help with positive ID for quite some time [8]. However, human identification becomes tricky and difficult when the cadaver is entirely burned and reduced to skeletal bones. Anthropological and dental examinations are particularly useful in these cases for determining a person's identification [9].

The mandible is the largest and most robust of the facial bones. It features a convex front and a horizontally curved body with two broad rami that rise behind it. In the rami, you can find the coronoid and condyloid processes. The coronoid process is a small, triangular bone plate that projects upward and slightly forward from the skull. Its anterior border penetrates the ramus, while its posterior border encloses the mandibular incisurae [10]. Attachments for the temporalis muscle can be found on its medial surface and margins [11]. Variations in morphology are the result of both hereditary factors and functional changes that occur during the embryonic period. The dynamic interaction between muscle and bone can potentially modify the morphology of the damaged bone [5].

Morphological variations, such as the shape of the condyle (which can be angled, round, convex, or flat), the width of the sigmoid notch, and the slant of the sigmoid fossa, have all been documented in the literature as being useful in anthropological and forensic investigations [5,6].

Variation in the shape of the coronoid process is associated with the attachment of the temporalis muscle and the chewing habits of the individual and may have hereditary or functional origins. A further anthropological use is as a race indicator. When the coronoid process is abnormally lengthy on one side of the face, it might cause difficulties with opening the mouth and moving the jaw [12]. Most of these studies to date have relied on mandibles that have been preserved in dry ice after being removed from deceased people's bodies [5,6]. It is helpful for oral and maxillofacial surgeons to be familiar with the many morphological types of the coronoid process. It is a great donor graft location for reconstructive facial procedures such as maxillary augmentation, mandibular fracture repair, and orbital floor reconstruction [6].

The mandibular condyle also varies greatly in appearance across different people and time periods. Osteology states that morphologic changes arise from both natural variation throughout development and the remodeling of the condyle to accommodate for things like malocclusion, trauma, and other disorders and illnesses that might affect development. Understanding the mandible's architecture and morphology is vital for differentiating between a healthy variant and a diseased state [13].

Comparing antemortem and postmortem radiographs is one of the quickest and most accurate human identification methods used in forensics [7]. In dentistry, panoramic pictures are the go-to radiograph for diagnosis and monitoring the efficacy of different treatments. It has also been asserted that panoramic radiography is reliable for depicting the condyle and evaluating temporomandibular joint problems [14]. Orthopantomographic maxillofacial radiography is less expensive than CT, MRI, and CBCT and is thus widely utilized as a routine screening technique in the diagnosis and treatment planning across many branches of dentistry. Oftentimes, the coronoid process, condyle, and sigmoid notch can be seen in a panoramic image, allowing for positive identification of a person whether they are alive or dead when antemortem records are provided and compared with postmortem records [15].

In this study, researchers used orthopanoramic pictures to evaluate the various forms of the coronoid process, condyle, and sigmoid notch. The participants in the study were split into four groups, each representing a 10-year age range. Different sexes and both sexes of the same individual were compared for morphological differences.

The condylar process is the primary anatomical feature of the mandible, and it is accountable for the development of the jaw in both the vertical and sagittal planes [15]. While our research showed that angular condyles were more common in older males, younger males, and all age groups, round condyles were more common in females. Consistent with our findings, Singh et al. found that the round/oval form was frequent in both sexes among the East Indian population [16]. In addition, the results demonstrate that all male instances were bilaterally angle-shaped, but female cases were more frequently spherical on the right side.

Similar to previous research by Shakya et al. and Tapas, the present study found that triangular coronoid processes were the most prevalent shape across all age groups and sexes [5,17]. Not only that, but the vast majority of cases were bilaterally triangular in shape, and this was true across both sexes. However, the current study contradicts the findings of Shakya et al., who found that the sloping forms of the sigmoid notch were the most typically observed, followed by the round and wide forms [5]. As a result, the majority of instances in both sexes were broader on both sides.

If the antemortem data are kept, our study's findings imply that panoramic photographs of the coronoid process, condyle, and sigmoid notch can be employed as a much simpler and faster method of identifying an individual, particularly in times of mass tragedies.

## Conclusions

Our research shows that using panoramic photos to portray the different morphologies of the coronoid process, condyle, and sigmoid notch can be a much simpler and faster method of identifying an individual, especially in the event of a mass disaster, so long as antemortem data are kept. The method of radiographic identification of individuals has recently gained prominence due to its efficacy. Radiographs like these can be invaluable in forensic dentistry, where they can help unearth previously hidden evidence if premortem records are retained. As a potential approach for individual identification among our population, panoramic radiographs were used to investigate the varying morphological forms of the coronoid process, condyle, and sigmoid notch.
